# Piezo1 in immune cells: from mechanosensation to immunoregulation

**DOI:** 10.3389/fimmu.2026.1827414

**Published:** 2026-06-09

**Authors:** Yuling Liang, Qiuxia Chen, Shuqiao Zhang, Ting Xiang, Juan Du, Shijun Zhang

**Affiliations:** 1 Department of Traditional Chinese Medicine, The First Affiliated Hospital, Sun Yat-sen University, Guangzhou, Guangdong, China; 2 The First Affiliated Hospital of Guangzhou University of Chinese Medicine, Guangzhou, Guangdong, China; 3 National Key Laboratory of Intelligent Tracking and Forecasting for Infectious Diseases, Beijing Ditan Hospital, Capital Medical University, Beijing, China; 4 Beijing Institute of Infectious Diseases, Beijing, China; 5 National Center for Infectious Diseases, Beijing Ditan Hospital, Capital Medical University, Beijing, China; 6 Beijing Key Laboratory of Viral Infectious Diseases, Beijing Ditan Hospital, Capital Medical University, Beijing, China

**Keywords:** cancer immunity, immune cells, immunotherapy, mechanoimmunology, Piezo1

## Abstract

**Background:**

The immune system is central to host defense against pathogens and the maintenance of organismal homeostasis. Immune cells often reside in or migrate through tissue microenvironments characterized by significant mechanical activity, where they are continuously subjected to diverse mechanical stimuli. Despite the ubiquity of mechanical signals in such contexts, the precise regulatory mechanisms by which these signals control immune cell function in a context-dependent manner remain incompletely understood.

**Main body:**

In recent years, the mechanosensitive ion channel Piezo1 has emerged as a core component of cellular mechanical sensing system, attracting considerable attention in immunology. This review integrates the latest research advances to systematically analyze the role of Piezo1 in both innate and adaptive immune cells. We discuss how Piezo1 is widely involved in key immune processes, including cell activation, differentiation, and functional regulation, and highlight the context-dependent nature of Piezo1 signaling, which can drive divergent immune outcomes depending on cellular lineage and pathological microenvironment.

**Conclusions:**

By synthesizing the current evidence, this review provides a theoretical foundation for understanding mechanical signaling in immunity. Crucially, this underscores that the functional duality and context-dependency of Piezo1 present both opportunities and challenges for drug targeting, offering insights for the development of precision immunotherapies that account for specific pathological settings.

## 1 Background

As the basic unit of living organisms, the behavior of cells is not only regulated by biochemical signals, but also profoundly guided by the physical microenvironment. Cells can sense and respond to physical stimuli, such as stiffness, fluid shear stress (FSS), osmotic pressure, and stretching of their surrounding matrix, and convert these mechanical signals into intracellular biochemical reactions, which is called mechanical transduction (1–4). This process is essential for cell proliferation, differentiation, migration, and fate determination and has been at the forefront of biomedical research in recent years.

The discovery of Piezo ion channels is a landmark in this field. As an important member of the mechanosensitive ion channel family, the Piezo family, including Piezo1 and Piezo2, was first discovered and named by Coste et al. in 2010, which is the first mammalian mechanosensitive cation channel identified (5). Among them, Piezo1 is widely expressed in a variety of tissues and cell types, with its large trimeric propeller-like structure, which is activated in direct response to changes in cell membrane tension, converting sensed mechanical stimuli into bioelectrical and biochemical signals (4, 6–11). Once opened, it allows the influx of various cations, such as Ca²^+^, Na^+^, and K^+^, of which the influx of Ca²^+^ is particularly critical and can act as an important second messenger to rapidly trigger a wide range of downstream signaling pathways (5, 10, 12, 13).

Notably, immune cells are in a highly dynamic microenvironment with varying mechanical properties throughout their life cycle (14). Immune cells are constantly exposed to complex mechanical force fields, from being subjected to blood flow shear force circulation in blood vessels to encountering extrusion and deformation when crossing blood vessel walls, patrolling tissues of different hardness, and interacting with other cells (15–18). Immune cells perform core functions of immune surveillance and host defense. The internal mechanism of accurate sensing of complex and variable mechanical signals has not been fully elucidated. In recent years, cross-studies of mechanobiology and immunology have revealed that Piezo1 plays a central role in regulating the activation, migration, and effector functions of immune cells by sensing mechanical signals and converting them into Ca^2+^ influx, and is a key molecule in mechanoimmune transduction (19–22). An in-depth understanding of the molecular mechanisms of Piezo1 in immune regulation is of great significance. On the one hand, it will expand our basic understanding of the interaction between mechanical force sensing and immune cells; on the other hand, it will provide a theoretical basis for the development of new immunotherapies targeting mechanosensitive channels.

## 2 Structural and functional properties of Piezo1

**Figure 1:**
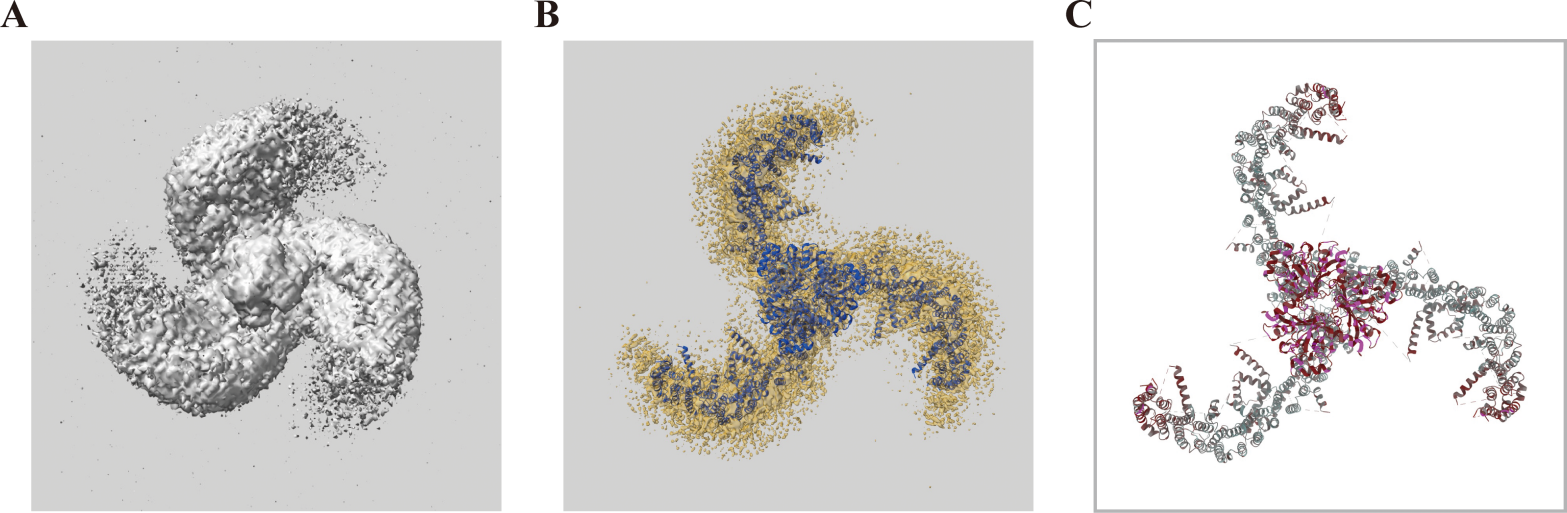
Cryo-EM structure of the Piezo1 protein. **(A)** 3D surface view of the cryo-EM density map along the Z-axis; **(B)** Overlay of the cryo-EM density map (transparent grey) and the fitted atomic model along the Z-axis; **(C)** Atomic model colored by Q-score per residue, viewed along the Z-axis. The cryo-EM density map was downloaded from the Electron Microscopy Data Bank (EMDB accession No. EMD-39205), and the atomic coordinates correspond to PDB ID: 8yez. Both EMDB and PDB data are publicly available under public domain.

Piezo1 is a mechanosensitive cation channel encoded by *PIEZO1* gene, with a molecular weight of approximately 286 kDa (5). The channel exists as a homotrimer and exhibits a unique three-leaf propeller-like spatial configuration (**Figures 1A–C**). Each monomer is composed of approximately 2500 amino acids and contains 24–38 transmembrane helices, which are organized into multiple structural repeat units and can be divided into two functional regions. The peripheral blade region senses membrane tension changes through special curvature structures, while the central pore region regulates selective cation permeability.

In the closed state, the pore has multiple narrow structures, including cytoplasmic “neck” restraints formed by the hydrophobic residues. Mechanical stimuli are delivered through the peripheral OARS and transmitted to the central pore via an intracellular lever (beam structure) that is approximately 90 Å long to induce channel opening. The C-terminal domain contains a functional motif rich in glutamate, which can bind to calmodulin and other signaling molecules to participate in gating regulation and downstream signal transduction. The extracellular N-terminal domain maintains membrane localization stability and mechanical sensitivity through glycosylation (23–27).

The molecular architecture of Piezo1, characterized by multi-domain coordination, facilitates efficient mechanoelectrical signal conversion. As a large trimeric mechanosensitive ion channel localized to the plasma membrane, its outward-extending blade-like peripheral regions serve as the primary force sensors. According to the force-from-lipids model, upon sensing mechanical stimuli, increased membrane tension flattens these curved blades, thereby opening the central pore (23, 28). This rapid conformational rearrangement allows Ca²^+^ to flood the cytoplasm, initiating downstream mechanotransduction cascades crucial for immune cell function. These unique structural features provide the structural basis for the context-specific functions of Piezo1 across distinct tissue microenvironments. The intrinsic mechanical properties of different tissues dictate the threshold and intensity of this structural rearrangement: In the vascular system, FSS, acting as a dynamic mechanical stimulus, activates Piezo1 by stretching the plasma membrane, triggering Ca²^+^ influx and CaMKII-NFAT signaling to regulate T cell activation and migration (29). Conversely, in pathologically stiffened environments such as fibrotic livers or solid tumors, elevated matrix stiffness is perceived via the peripheral blades of Piezo1, inducing sustained Ca²^+^ oscillations. This not only drives metabolic reprogramming toward M1 polarization in macrophages but also promotes the secretion of specific cytokines by dendritic cells (DCs), thereby determining the differentiation fate of T cells toward either Th1 or Treg cell phenotypes (30–32). Furthermore, in the relatively compliant environment of lymphoid tissues, Piezo1-mediated perception of membrane-bound antigens by B cells relies on the fine-tuned response of their structure to membrane tension, consequently influencing antibody class switching (22, 33).

**Figure 2:**
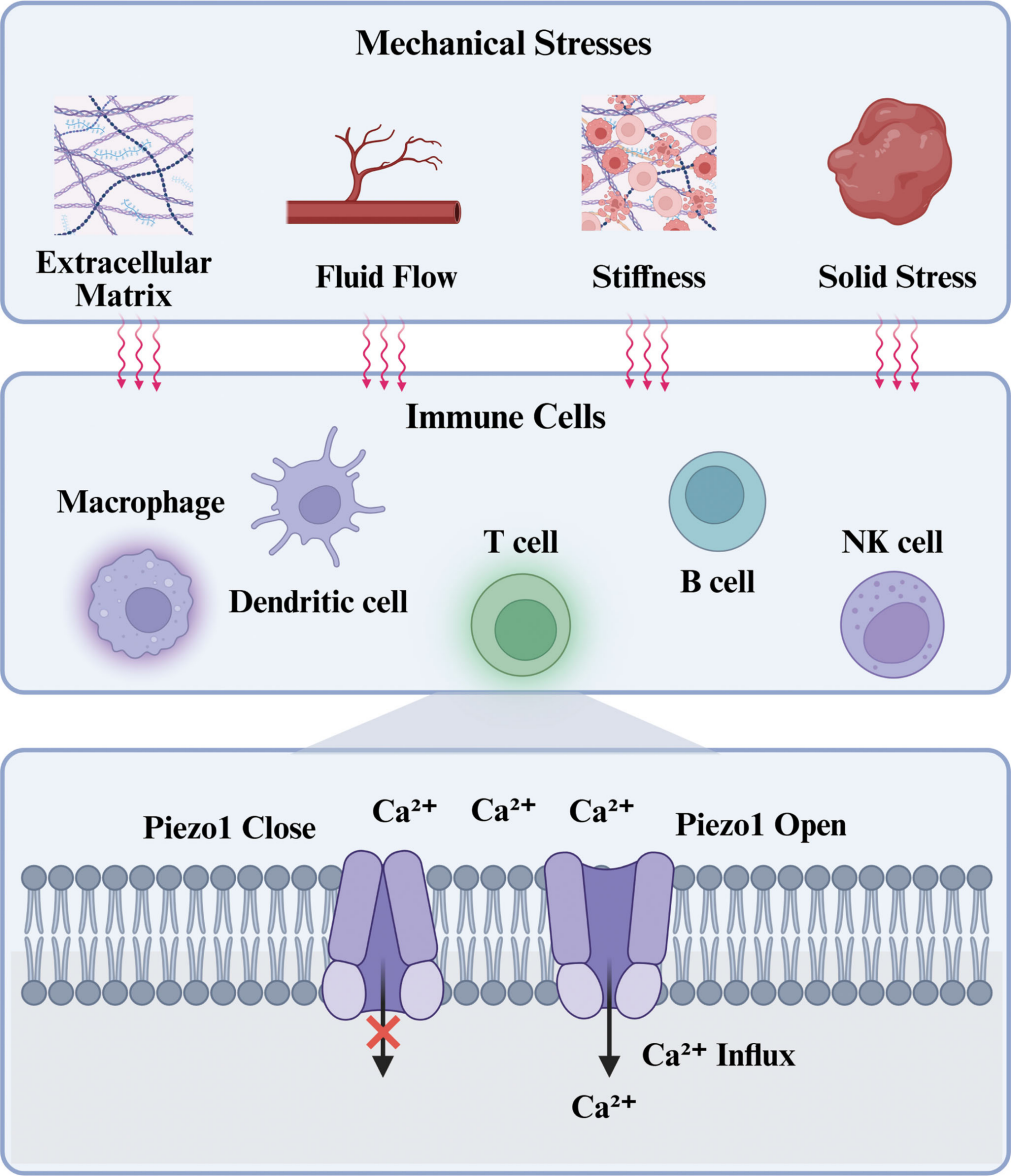
Transduction of mechanical stresses sensed by immune cells via the Piezo1 ion channel. Top panel: Physiological and pathological conditions impose various forms of mechanical stress on immune cells. These forces encompass mechanical cues derived from extracellular matrix architecture, shear stress generated by fluid flow, substrate stiffness, and solid stress arising from tissue compression, among others; Middle panel: Innate immune cells (macrophages, dendritic cells, NK cells) and adaptive immune cells (T cells, B cells) are continuously exposed to mechanical signals; Bottom panel: Piezo1, localized to the plasma membrane of immune cells, functions as a key mechanosensor. Upon mechanical stimulation, Piezo1 undergoes a conformational change from a closed to an open state, facilitating Ca²^+^ influx. This process converts mechanical forces into biochemical signals that regulate diverse immune cell functions.

In conclusion, by decoding mechanical forces of varying amplitudes and types through its structure, Piezo1 extensively regulates the activation, polarization, migration, and effector functions of immune cells, including DCs, macrophages, T cells, and B cells. This enables precise, differential biochemical signaling output tailored to specific tissue physical properties within the same immune system, establishing Piezo1 as a central hub connecting the mechanical microenvironment to immune responses (**Figure 2**).

## 3 Functional regulation of Piezo1 in innate immune cells

### 3.1 DCs

#### 3.1.1 Regulation of Piezo1-mediated stiffness sensing on DC activation and function

Tissue stiffness undergoes dynamic changes under both physiological and pathological conditions. For instance, in fibrotic diseases and solid tumors, the extracellular matrix often becomes abnormally stiff. Studies have demonstrated that changes in the mechanical stiffness of the tissue microenvironment exert direct and profound effects on DCs. When cultured on substrates mimicking the stiffness of physiological resting tissues, DCs exhibit relatively weak capabilities in terms of proliferation, activation, and cytokine production. In contrast, when placed on high-stiffness substrates that simulate a pathological microenvironment, DCs show a sustained increase in glucose metabolic flux and display a more activated state, thereby enabling the initiation of an effective adaptive immune response (34). These results establish mechanical stiffness as an essential microenvironmental determinant of DC activation and function while identifying Piezo1 as a pivotal mechanosensory component in this regulatory pathway.

#### 3.1.2 DCs regulate T cell differentiation fate via Piezo1-mediated cytokine balance

Wang et al. revealed that Piezo1 on DCs, upon sensing mechanical stiffness or inflammatory signals, directs reciprocal differentiation of Th1 and Treg cells. This mechanism has been primarily elucidated in the type 1 conventional dendritic cell (cDC1) subset, which specializes in cross-presenting antigens to CD8^+^ T cells. Specifically, genetic deletion of Piezo1 in DCs suppressed Th1 cell generation while promoting Treg cell development, ultimately accelerating tumor growth in mice. Mechanistically, the loss of Piezo1 disrupted the balance between the secretion of the polarizing cytokines TGF-β1 and IL-12. This imbalance enhances TGFβR2-p-Smad3 signaling, thereby promoting Treg cell differentiation and attenuating IL-12Rβ2-p-STAT4 signaling, consequently inhibiting Th1 cell differentiation. Further investigation revealed that Piezo1 integrates the SIRT1-HIF1α-dependent metabolic pathway with the Ca^2+^-calcineurin-NFAT signaling axis, enabling the precise regulation of Th1 and Treg cell lineage commitment by coordinating DC-derived IL-12 and TGF-β1 (30).

Nevertheless, the loss of Piezo1 in DCs does not uniformly drive tumor progression. However, a study focusing on tumor-infiltrating CD11b^+^ DCs reached contrasting conclusions. This study found that conditional knockout of Piezo1 in CD11b^+^ DCs significantly suppressed both primary and metastatic tumor burden. Further analysis revealed that Piezo1 deficiency increased the infiltration of CD11b^+^ DCs into tumors, and that these DCs underwent transcriptional changes associated with enhanced antigen presentation and improved T cell activation. Specifically, within the CD11b^+^ DC population, the type 2 conventional dendritic cell (cDC2A) subset, which specializes in the activation of CD4^+^ T cells, exhibits a significant enhancement in its capacity for cross-activation of CD8^+^ T cells in the absence of Piezo1. This study represents the first report linking mechanical sensation to the cross-presentation function in cDC2A cells, suggesting that Piezo1 may exert distinct or even opposing functions, depending on the DCs subset or specific pathological context (35).

#### 3.1.3 Strategies targeting Piezo1 to enhance DC-based immunotherapy

Based on the mechanistic understanding of Piezo1, researchers have explored its application in immunotherapy. One study developed a personalized mineralized vaccine named Cell@CaP, which actively triggers the Piezo1 channel in DCs upon interaction by leveraging its high mechanical stiffness, thereby inducing Ca²^+^ influx. This mechanical stimulation synergizes with vaccine-released cGAMP (a STING agonist) and tumor antigens, collectively activating multiple signaling pathways, including cGAS-STING and PI3K-Akt. Consequently, it robustly promotes DCs maturation and the secretion of the proinflammatory cytokines IFN-β and TNF-α, effectively suppressing tumor recurrence and metastasis in a post-surgical model (36).

Similarly, another study on cellular backpack technology underscores the critical role of Piezo1. This revealed that the mere physical attachment of these backpacks to the DCs surface, regardless of whether they carry antigens, is sufficient to induce DCs maturation by opening the Piezo1 channels. This mechano-immunity drives DCs to produce type I interferons and remodel their cytoskeleton, which subsequently synergizes with radiotherapy to generate potent antitumor effects (31). This finding further validates the potential of targeting the Piezo1 pathway to enhance the efficacy of DC-based vaccines.

Although Piezo1 functions as a “double-edged sword” in DC subsets, both technologies successfully steer their function toward a unified anti-tumor mode by imposing defined mechanical stiffness. The underlying common mechanism lies in the forced activation of Piezo1 via high−stiffness physical stimuli, which triggers sustained Ca²^+^ influx and synergizes with critical pathways such as cGAS−STING and type I interferon to fundamentally remodel DC antigen presentation and T cell priming. This robust, pro−inflammatory activation state effectively overrides suppressive signals within the tumor microenvironment (TME), transforming Piezo1 role from a physiological “fine−tuning modulator” into a therapeutic “immune accelerator.” By ultimately focusing on enhancing cytotoxic T cell responses, this strategy circumvents the risk of immunosuppression arising from dysregulated Piezo1 activity. Consequently, precise physical control over Piezo1 activation is pivotal for translating it from a “double−edged sword” into a potent weapon for immunotherapy.

### 3.2 Macrophages

#### 3.2.1 Piezo1 as a mechanosensor and functional trigger in macrophages

Macrophages infiltrate various tissues, and the physical properties of their microenvironment, such as tissue stiffening in hepatic and pulmonary fibrosis, stiffness of atherosclerotic plaques, and mechanical loading during bone regeneration, are critical factors that influence their behavior. Studies have shown that when macrophages are cultured on rigid substrates, the expression and activity of Piezo1 is significantly upregulated, accompanied by enhanced Ca²^+^ influx (37). Conversely, inhibition of Piezo1, whether by culturing on soft substrates, genetic knockout, or pharmacological inhibitors (e.g., GsMTx4), compromises macrophage response to stiffness stimulation (38, 39). This indicates that Piezo1 is essential for stiffness sensing in the macrophages. Furthermore, mechanical forces such as static and cyclic stretching can modulate macrophage responses to mechanical signals by regulating the crosstalk between Piezo1 and integrin CD11b, thereby influencing the macrophage morphology and functional responses (40). Thus, Piezo1 serves as a central molecule for macrophages to perceive diverse mechanical signals, and its activation acts as a critical switch that triggers subsequent functional changes in the macrophages.

#### 3.2.2 The dual role of Piezo1 in macrophage polarization

Macrophages can polarize into proinflammatory M1 or anti-inflammatory and reparative M2 phenotypes, a process precisely regulated by Piezo1.

##### 3.2.2.1 Promotion of M1 polarization and proinflammatory responses

In various disease models, activation of Piezo1 tends to drive macrophages toward the M1 phenotype. For instance, in models of bone infection, psoriasis, temporomandibular joint arthritis, and spinal cord injury, Piezo1 activation promotes the secretion of proinflammatory cytokines, such as TNF-α and IL-1β, via signaling pathways such as Ca²^+^-CaMKII-NF-κB, thereby exacerbating local inflammation (41–44). In fungal keratitis and osteoarthritis, Piezo1 further enhances M1-associated immune responses by activating the pyrin inflammasome and promoting glycolysis (45, 46).

##### 3.2.2.2 Induction of M2 polarization and reparative functions

However, in specific tissue repair contexts, Piezo1 can promote M2 polarization. For example, mechanical stretching activates Piezo1, leading to p53 deacetylation in macrophages, which promotes their transition to the M2 phenotype. These M2 macrophages secrete TGF-β1, thereby facilitating the osteogenic differentiation of bone marrow mesenchymal stem cells and promoting bone formation (47, 48). In a masticatory mucosa model, chewing stress-induced Piezo1 activation drives macrophage M2 polarization, increases TGF-β1 secretion, and accelerates wound healing (49). Furthermore, low-stiffness materials such as Ti2448 alloy and specific nanostructured PEEK implants promote a shift toward the M2 phenotype by inhibiting Piezo1 activity, creating an anti-inflammatory microenvironment that enhances osseointegration (50–52).

Piezo1 exerts a bidirectional regulatory effect on macrophage polarization, a mechanism closely linked to mechanical signals and inflammatory microenvironment. Under conditions of high substrate stiffness or a proinflammatory milieu, Piezo1 activation promotes macrophage polarization toward the proinflammatory M1 phenotype via pathways such as YAP or NF-κB, while concurrently suppressing M2-associated pathways, including STAT6. Conversely, on soft substrates or upon pharmacological inhibition of Piezo1, suppression of the STAT6 signaling axis is alleviated, favoring macrophage polarization toward the anti-inflammatory M2 phenotype. This shift subsequently modulates immune responses and influences tissue repair and inflammation (39, 43). Thus, distinct mechanical stimuli and inflammatory microenvironments engage divergent downstream signaling pathways via Piezo1, ultimately determining the direction of macrophage polarization.

#### 3.2.3 Key regulatory roles of Piezo1 in core macrophage functions

Piezo1 not only regulates macrophage polarization phenotypes, but also critically governs the execution of core physiological functions, including phagocytic clearance, metabolic reprogramming, and crosstalk with Toll-like receptor (TLR) signaling pathways (**Table 1**).

**Table 1:** Multidimensional mechanisms and signaling pathways of Piezo1 in regulating macrophage functions.

Core functions	Specific mechanisms and actions	Key signaling pathways	Related physiological/pathological models
Phagocytosis	Enhance phagocytosis and bactericidal activity against fungi/bacteria	Pyrin inflammasome–CaMKII-Mst1/2-Rac axis	Fungal keratitis, bacterial infections (45, 53)
	Promote clearance of apoptotic cells to ensure lysosomal acidification and degradation	Piezo1-Ca²^+^-lysosomal acidification	Liver fibrosis (high-stiffness microenvironment) (54)
	Participate in red blood cell renewal and iron metabolism regulation	Piezo1-Ca²^+^-iron metabolism axis	Red blood cell renewal (55)
Metabolic Reprogramming	Drive metabolism toward aerobic glycolysis to provide energy and precursors	CaMKII-HIF1α-glycolysis axis	Matrix stiffness, hydrostatic pressure stimulation (56, 57)
	Upregulate glycolytic genes (Glut1, Aldoa) and enhance glycolytic activity	Piezo1-Ca²^+^-Glut1/Aldoa upregulation	Mechanical microenvironment stimulation (56, 57)
Crosstalk with TLR Signaling	Synergize with TLR4 to enhance bactericidal function	TLR4-Piezo1-CaMKII-Mst1/2-Rac axis	Sepsis, bacterial infections (53)
	Upregulate Piezo1 via SGK1 to inhibit TLR4-NFκB and induce immunosuppression	SGK1-Piezo1-TLR4-NFκB inhibition axis	Sepsis (high-dose dexamethasone) (58)
	Promote TLR9 endosomal translocation and enhance CpG-ODN-induced inflammation	Piezo1-CaMKII-NF-κB-TLR9 axis	Acute liver failure (59)

##### 3.2.3.1 Phagocytosis

Piezo1 is a critical factor in enhancing the phagocytic capacity of macrophages. In models of fungal keratitis and bacterial infection, the activation of Piezo1 increases Ca²^+^ influx, which markedly enhances macrophage phagocytosis of pathogens and bactericidal activity by triggering the pyrin inflammasome or activating the CaMKII-Mst1/2-Rac axis (45, 53). In liver fibrosis, the activation of Piezo1 not only enhances the ability of macrophages within a high-stiffness microenvironment to clear apoptotic cells but also promotes the acidification of phagolysosomes to ensure efficient degradation of engulfed material, thereby facilitating the resolution of inflammation (54). Moreover, by modulating macrophage phagocytic function, Piezo1 is involved in erythrocyte turnover and influences systemic iron metabolism (55).

##### 3.2.3.2 Metabolic reprogramming

Macrophage immune functions are closely related to the metabolic state. Research has identified Piezo1 as a key mechanosensor and regulator of metabolic reprogramming in macrophages. Mechanical stimuli, such as substrate stiffness, hydrostatic pressure, or tissue stretch, activate Piezo1and trigger Ca^2+^ influx. This activation initiates the CaMKII-HIF1α signaling axis, driving a shift in macrophage metabolism toward aerobic glycolysis. Concurrently, it upregulates the expression of glycolysis-related genes such as Glut1 and Aldoa, and enhances glycolytic activity (56, 57). This metabolic reprogramming supplies the energy and biosynthetic precursors necessary for macrophage effector functions, thereby augmenting the inflammatory response, promoting the secretion of inflammatory cytokines, and facilitating immune-cell infiltration. Conversely, the inhibition of Piezo1 impairs both glycolytic and inflammatory responses. These findings indicated that Piezo1 regulates the immune function of macrophages in mechanical microenvironments by modulating their metabolic state.

##### 3.2.3.3 Crosstalk with TLR signaling

Piezo1 interacts significantly with classical pathogen-recognition pathways. Research has demonstrated that lipopolysaccharide promotes the assembly of Piezo1-TLR4 complexes through TLR4 signaling, thereby enhancing Piezo1-mediated Ca²^+^ influx and synergistically improving macrophage bactericidal function via the CaMKII-Mst1/2-Rac axis (53). Conversely, in sepsis models, high-dose dexamethasone upregulates Piezo1 through serum or glucocorticoid-regulated kinase 1, which subsequently suppresses TLR4-NF-κB p65 activation and produces an immunosuppressive effect (58). In acute liver failure, Piezo1 promotes TLR9 endosomal translocation, enhancing CpG-ODN-induced CaMKII-NF-κB signaling and consequently increasing proinflammatory cytokine production (59).

The key to explaining these seemingly paradoxical phenomena lies in the structural dynamics of Piezo1 and cell type-specific signaling decoding mechanisms. First, the three-dimensional (3D) conformation of Piezo1 possesses a multimodal response potential; its blade-like peripheral regions, acting as force sensors, undergo varying degrees of conformational bending or unfolding under different pathological microenvironments, depending on membrane tension and interacting proteins, thereby activating distinct downstream branches (23, 28). Second, competitive resource allocation and spatiotemporal decoding of Ca²^+^ signaling within downstream networks are critical: Piezo1 co-assembles with TLR4 to synergistically enhance bactericidal function via the CaMKII-Mst1/2-Rac axis (53), whereas in the dexamethasone-SGK1 model, Piezo1 upregulation alters the spatiotemporal characteristics of Ca²^+^ signaling, potentially inhibiting NF-κB nuclear translocation via calcineurin activation (58); in the CpG-ODN-TLR9 model, Piezo1 acts as a molecular chaperone to facilitate TLR9 endosomal translocation, thereby enhancing specific signaling (59). Therefore, the interaction between Piezo1 and TLR signaling represents a context-dependent decoding based on pathogen nature and tissue microenvironment. Although this structural plasticity allows Piezo1 to serve as a fine-tuning node for innate immune intensity, it also underscores the necessity of strictly considering specific disease contexts in targeted therapies.

#### 3.2.4 Novel therapeutic strategies targeting macrophage Piezo1 in disease

##### 3.2.4.1 Fibrotic diseases: bidirectional intervention based on disease stage stratification

In hepatic and renal fibrosis models, Piezo1 activation drives pathological progression via a dual mechanism: On one hand, Piezo1-mediated Ca²^+^ influx activates calpain proteases, promoting LAMP1 cleavage and cathepsin S secretion, thereby remodeling the immune microenvironment (32); on the other hand, Piezo1 enhances macrophage infiltration and M1 polarization by modulating inflammatory pathways such as CCL2−CCR2, Notch, and NF−κB, ultimately facilitating EMT and collagen deposition (60). Notably, Piezo1 exhibits functional heterogeneity across distinct stages of fibrosis, while inhibiting its activity significantly alleviates fibrotic lesions at advanced stages. Pharmacological activation of Piezo1 using Yoda1 in murine models of liver fibrosis accelerates inflammation resolution and fibrosis reversal by enhancing macrophage efferocytosis (54).

Therefore, intervention strategies targeting Piezo1 must be strictly tailored to the stage stratification of the disease. Regarding treatment timing, inhibition is warranted during the progressive phase to block pathogenic signaling axes and mitigate inflammation and collagen deposition. Conversely, activation may be considered during the inflammation resolution and tissue repair phases to enhance regeneration. In terms of targeted delivery, given that the traditional Chinese medicine component lithospermic acid has been validated for its anti-fibrotic effects via Piezo1 inhibition, utilizing its chemical structure as a pharmacophore for structural optimization or conjugation with macrophage-targeting moieties warrants further investigation as a potential therapeutic approach (61). However, this strategy presents certain side effects and limitations. For safety, since Piezo1 confers protective functions during the resolution phase, systemic long-term inhibition may interfere with tissue remodeling. Existing botanical compounds generally suffer from low bioavailability and insufficient target specificity, and the lack of precise methods to discriminate fibrosis stages and macrophage phenotypes currently restricts their clinical translation.

##### 3.2.4.2 Vascular diseases: pathological context-specific precision blockade

In the treatment of vascular diseases, Piezo1 exhibits high pathological context specificity, necessitating caution against the risks of blind intervention that ignores pathological heterogeneity. In atherosclerosis, macrophage Piezo1 senses oxLDL and environmental stiffness signals to promote lipid uptake and foam cell formation. Thus, inhibition of its activity is required to curb plaque progression (38, 62, 63). Indeed, the traditional Chinese medicine component salvianolic acid B and the herbal formula Guizhitongluo Tablet have been validated for their anti-atherosclerotic effects via Piezo1 inhibition (64, 65). However, in models of hindlimb ischemia, matrix stiffness-activated Piezo1 suppresses FGF2 secretion via the CaMKII-ETS1 pathway. Consequently, inhibiting Piezo1 under these conditions impairs angiogenesis and perfusion recovery (66). Therefore, the timing of treatment must be strictly differentiated based on specific pathological background. Regarding potential side effects, since Piezo1 is widely involved in vascular mechanoperception, systemic intervention may disrupt vascular tone regulation and hemodynamic homeostasis. There is currently a lack of reliable *in vivo* methods to distinguish between plaque formation and ischemic stages; this deficiency easily leads to misjudgment of the pathological phase, resulting in erroneous intervention directions that may exacerbate the disease.

##### 3.2.4.3 Intervention strategies for infection and inflammatory injury

Piezo1 modulators have specific applications in the treatment of infection and acute injury. Regarding treatment timing, agonists such as Yoda1 are suitable for scenarios requiring enhanced immune clearance or promotion of tissue repair, as seen in fungal keratitis (45), the inhibitor GsMTx4 is indicated for stages necessitating the suppression of excessive inflammatory responses. Mechanistically, GsMTx4 promotes M2 polarization of macrophages by inhibiting Piezo1-mediated mechanosignaling, thereby mitigating hyperinflammation and tissue damage in malaria-associated acute lung injury and experimental cerebral malaria (67). This suggests that potential side effects and limitations lie in the risk of tissue damage due to excessive immune activation if agonists are administered during non-infectious periods, whereas inappropriate use of inhibitors may compromise the host pathogen clearance. Therefore, future drug development targeting Piezo1 requires a strict delineation of the therapeutic window to balance the benefits and risks of its immunomodulatory effects.

### 3.3 Natural killer cells

Research on the function and role of Piezo1 in Natural killer (NK) cells remains limited and represents a promising direction for future research. Current evidence indicates that Piezo1-mediated mechanosensing serves as a central hub for NK cells to effectively execute their cytotoxic function within 3D physiological environments. Studies have revealed that the intrinsic stiffness of target cells is a critical determinant of NK cell killing efficiency. Pharmacological softening of tumor cells significantly impairs the clearance capacity of NK cells in a 3D collagen matrix. Conversely, artificially increasing tumor cell stiffness substantially enhances the NK cell-killing efficiency and accelerates target elimination and disengagement. The key mechanism underlying this phenomenon is that the perception of external mechanical signals by NK cells is primarily mediated by the mechanosensitive ion channel Piezo1. Gain- and loss-of-function experiments have demonstrated that inhibiting Piezo1 with GsMTx4 completely abrogates the ability of NK cells to respond to targets of varying stiffness, severely compromising both their killing efficiency and capacity to infiltrate the 3D matrix. In contrast, specific activation of Piezo1 with the agonist Yoda1 enhances NK cell cytotoxic potency and promotes their infiltration (68).

Although NK cells are well documented to exhibit remarkable functional plasticity in response to cytokine and tissue microenvironmental signals, the precise role of Piezo1 in regulating this heterogeneity remains poorly defined. Current evidence indicates that mechanical signals can modulate NK cell cytotoxicity and migratory capacity. However, compared with their well-established functions in T cells or dendritic cells, the contribution of Piezo1 to specific NK cell functional states, particularly its role in the transition between cytotoxic phenotypes and exhausted or tolerant states, remains largely uncharacterized. Given the paucity of direct studies on Piezo1 in NK cells, this review refrains from extensive speculation. Future investigations should focus on elucidating whether Piezo1 mediated mechanotransduction facilitates NK cell adaptation to diverse tissue microenvironments. Such endeavors will not only revel how mechanical cues govern NK cell immune surveillance, but may also establish Piezo1 as a novel target for immunotherapy.

## 4 Functional regulation of Piezo1 in adaptive immune cells

### 4.1 T cells

#### 4.1.1 Regulatory mechanisms of Piezo1 in T cell activation and proliferation

Effective activation of T cells is a prerequisite for clonal expansion and immune function. Substantial evidence indicates that Piezo1 serves as a critical mechanosensor and is essential for effective T cell receptor (TCR) activation. By perceiving physical cues from the microenvironment and converting them into biochemical signals, Piezo1 precisely orchestrates T cell activation.

##### 4.1.1.1 As a specialized mechanosensor in the immunological synapse

T cell activation requires not only the recognition of peptide-major histocompatibility complex (pMHC) on antigen-presenting cells (APCs) by the TCR but also relies on mechanical forces. During immune synapse formation, the interaction between TCR and pMHC generates mechanical pulling forces that alter local membrane tension. Piezo1 serves as the central receptor for sensing mechanical force. Increased membrane tension activates Piezo1, triggering Ca²^+^ influx and subsequent calpain activation. By remodeling the cortical actin cytoskeleton and optimizing the assembly of TCR signaling modules, calpain directly converts mechanical forces into enhanced biochemical signal strength (69, 70).

##### 4.1.1.2 FSS-mediated enhancement of T cell activation

The FSS in the circulatory system represents a typical physiological mechanical force, wherein Piezo1 functions as a critical signal amplifier. FSS enhances T cell activation in a Piezo1-dependent manner. Studies have indicated that when combined with CD3 and CD28 stimulation, FSS significantly enhances the phosphorylation or activation of key signaling proteins, such as ZAP70, NFAT, NF-κB, and AP-1, and promotes the production of cytokines, including TNF-α, IL-2, and IFN-γ. When Piezo1 was inhibited by GsMTx4 or knocked down, this mechanical enhancement effect was significantly attenuated, whereas the Piezo1 agonist Yoda1 restored the T cell activation capacity. These findings identify Piezo1 as a central mediator governing the positive feedback regulation of T cell activation by physiological mechanical forces (29).

##### 4.1.1.3 The impact of TME mechanical stress on T cell activation

In pathological settings, the perception of TME mechanical stress by Piezo1 dictates T cell activation outcomes. Previous studies have demonstrated that in tumor-bearing mouse models, T cell-specific knockout of Piezo1 (P1KO) results in anti-tumor immune deficiency, which is characterized by accelerated tumor growth and unresponsiveness to therapy. Mechanistically, Piezo1 deficiency impairs the ability of CD4^+^ T cells to sense mechanical forces, resulting in a failure to provide effective helper signals and leading to a dysfunctional CD25^low^ PD−1^hi^ phenotype in CD8^+^ T cells. This demonstrates that Piezo1 is an essential sensor required to achieve full T cell activation under the physical barriers and mechanical stress of solid tumors (71).

#### 4.1.2 Regulation of T cell subset differentiation and function by Piezo1

Functioning as a key mechanosensor, the Piezo1 channel precisely governs the differentiation trajectory of distinct T cell subsets through Ca²^+^ influx in response to mechanical microenvironmental cues, thereby activating complex downstream signaling cascades.

##### 4.1.2.1 Regulation of helper T cell subsets

Th1 Differentiation: The regulation of Th1 differentiation by Piezo1 exemplifies how physical factors, such as matrix stiffness and shape immune balance. Studies indicate that Piezo1 deficiency in dendritic cells disrupts the SIRT1−HIF1α metabolic pathway and the Ca^2+^−calcineurin−NFAT signaling axis, leading to an imbalanced secretion of IL−12 and TGF−β1. This physical−biochemical coupling within the cytokine environment ultimately suppresses Th1 differentiation and promotes Treg formation (30).

Th17 Differentiation: The regulatory role of Piezo1 in Th17 differentiation is multilayered and context-specific. In psoriasis models, Piezo1 in keratinocytes senses local inflammatory mechanical signals to activate NF−κB, thereby promoting IL−23 production and indirectly driving the differentiation of naïve CD4^+^ T cells toward a Th17 phenotype (72). Conversely, in experimental autoimmune encephalomyelitis (EAE) models, T cell−intrinsic Piezo1 deficiency does not directly impair Th17 differentiation capacity but indirectly mitigates pathological damage by increasing the proportion of Tregs, suggesting that Piezo1 modulates Th17 function through microenvironmental remodeling (20, 73).

Th9 Differentiation: Piezo1 positively regulates Th9 differentiation through metabolic reprogramming, a downstream effector of mechanotransduction. In CD4^+^ T cells, Piezo1 deficiency enhances mitochondrial SIRT3−SDHA−dependent oxidative phosphorylation, suppresses HIF1α activity, and relieves the inhibition of the HIF1α−IL−9 axis, thereby exerting positive regulatory control on Th9 cell differentiation. This highlights the unique capacity of Piezo1 to convert mechanical signals into metabolic directives that determine the T cell differentiation fates (74).

##### 4.1.2.2 Regulation of Treg cells

Piezo1 exerts definitive cell-autonomous inhibition of Treg cells. In experimental autoimmune encephalomyelitis (EAE) models, T cell-specific deletion of Piezo1 leads to a significant increase in the proportion of Treg cells and disease amelioration by enhancing TGF−β signaling. Notably, the conditional ablation of Piezo1 specifically within Treg cells also results in marked disease attenuation, establishing Piezo1 as an intrinsic mechanical checkpoint that restricts Treg-mediated immune suppression and promotes inflammatory responses (20, 73). Importantly, this inhibitory effect is characterized by cell-context specificity, as Piezo1 deletion in DCs may indirectly paradoxically promote Treg differentiation (30).

##### 4.1.2.3 Role in T cell exhaustion

Under persistent mechanical stress in chronic infections and in the TME, Piezo1 emerges as a key driver of CD8^+^ T cell dysfunction. Within the mechanical landscape of the TME, terminally exhausted CD8^+^ T cell subsets upregulate Odd−skipped related transcription factor 2 (Osr2) expression via TCR signaling and the Piezo1-Ca²^+^-CREB axis. Osr2 subsequently drives cells toward terminal exhaustion by recruiting HDAC3 to epigenetically silence effector genes while concurrently upregulating inhibitory receptors, such as PD−1 and Tim−3 (75). This identifies the Piezo1−Osr2 axis as a crucial mechano−epigenetic coupling mechanism by which TME physical properties dictate T cell dysfunction. However, Zhang et al. offered an alternative perspective, proposing that Piezo1 is upregulated in activated cytotoxic T lymphocytes to negatively regulate cytotoxicity, and that Piezo1 inhibition can potentiate killing capacity (76).

#### 4.1.3 Regulatory mechanisms of Piezo1 in T cell migration and tissue homing

The precise migration of T cells to lesion sites is essential for their immune function, a process that relies on the perception of mechanical cues from the microenvironment. Piezo1 is the core molecular component of the mechanosensing mechanism. By integrating mechanical forces with biochemical signals, Piezo1 regulates chemotactic migration, integrin-dependent adhesion, and cytoskeletal dynamics in T cells, thereby influencing tissue-homing efficiency.

##### 4.1.3.1 Regulation of integrin-dependent chemotactic migration in T cells

When T cells respond to chemokines, adhesion plaques form at their leading edges, generating a local membrane tension. These mechanical cues guide mechanosensor Piezo1 to accumulate at the front. Subsequently, the activated Piezo1 triggers Ca²^+^ influx and calpain activation. This signaling pathway is essential for recruiting integrin LFA-1 to the leading edge, thereby reinforcing adhesion and driving directional movement. Consequently, Piezo1 deficiency disrupts local mechanosensing-dependent “outside-in” signaling, impairing integrin-dependent migration of T cells (21). Further studies indicated that the regulatory function of Piezo1 is closely associated with its spatial distribution. During chemotactic migration, Piezo1 and actin exhibit pronounced anterior-posterior axial polarity in T cells (77). This polarized distribution ensures that Piezo1 responds more effectively to mechanical signals at the leading edge and coordinates cytoskeletal reorganization, representing the spatial basis for the precise regulation of integrin-dependent chemotaxis by Piezo1.

##### 4.1.3.2 Impact on T cell tissue retention and persistence

Piezo1 influences not only the migratory capacity but also the tissue localization and survival of T cells. As a mechanosensitive Ca²^+^ channel that is widely expressed in CD4^+^ T cells, Piezo1 plays a critical role in their persistence. Studies have demonstrated that in multiple adoptive transfer-induced, T cell-mediated inflammatory models, including experimental autoimmune encephalomyelitis, inflammatory bowel disease, and graft-versus-host disease, CD4^+^ T cells lacking Piezo1 fail to effectively induce the disease, despite exhibiting enhanced proinflammatory cytokine secretion capability *in vitro*. This phenomenon is associated with impaired ability to establish and maintain a stable pool of effector memory T cells (CD44^hi^CD62L^low^). Co-transfer models further confirmed that this defect originates from an intrinsic reduction in the fitness and persistence capacity of Piezo1-deficient T cells. This suggests that Piezo1 plays a key role in regulating T cell homing to tissues, their residency within inflammatory environments, and their long-term survival, thereby influencing immunopathological functions (73).

##### 4.1.3.3 Dual roles in T cell tumor immunity

Research indicates that the function of Piezo1 during T cell recruitment to tumor sites differs from its role within the TME. During T cell migration toward tumor sites, Piezo1 plays a significant promoting role. As a crucial mechanosensor, Piezo1 is essential for integrin-dependent migration of T cells; its deficiency directly impairs their directional motility, thereby affecting their recruitment efficiency to the lesion site (21). However, when T cells successfully infiltrate the TME and are persistently exposed to unique mechanical and biochemical stimuli, Piezo1 undergoes a functional shift and exerts inhibitory effects. Studies have found that in activated and exhausted cytotoxic T cells, Piezo1 expression is further upregulated, and its function resembles that of a mechanical immune checkpoint. Inhibiting this activity, rather than completely deleting Piezo1, can significantly enhance the *in vitro* killing capacity and *in vivo* tumor infiltration efficiency of T cells, while also slowing tumor growth. This enhancement was achieved by increasing the cellular traction force and promoting F-actin assembly through pathways such as GRHL3-RNF114 (76). Consequently, Piezo1 plays a distinct dual role in T cell-mediated anti-tumor immunity.

Consequently, targeting strategies directed at Piezo1 must exhibit high contextual sensitivity to achieve spatiotemporally specific functional modulation (**Table 2**). During the initial phase of T cell infiltration into tumor tissues, the focus should be on preserving or enhancing the mechanotransductive function of Piezo1. Potential strategies include localized activation of mechanosignaling pathways using Piezo1 agonists or upregulation of Piezo1 expression in T cells via genetic engineering, thereby augmenting T cell motility and the capacity to penetrate dense matrices and overcoming physical barriers to improve homing efficiency into the tumor interior. Conversely, for T cells that have successfully infiltrated the tumor parenchyma, emphasis should shift to inhibiting the “immunological brake” function of Piezo1. Potential strategies involve utilizing specific small-molecule inhibitors of Piezo1 to block its ion channel activity or employing RNA interference and PROTAC technologies to specifically degrade Piezo1 protein in tumor-infiltrating lymphocytes. These approaches aim to relieve the inhibition of cytoskeletal remodeling and cytotoxic function, thereby rejuvenating the exhausted T cells. Such a staged and differential intervention strategy holds promise for optimizing the therapeutic window for Piezo1 targeting, although rigorous clinical validation is required.

**Table 2:** Therapeutic strategies for targeting Piezo1 in different functional stages.

Intervention stage	Target function	Intervention direction	Potential strategies	Expected effects
Infiltration phase	Promote migration	Activation/Upregulation	1. Pharmacological Activation: Local administration of Piezo1 agonists (e.g., Yoda1). 2. Genetic Enhancement: Utilizing CRISPRa or overexpression vectors to upregulate Piezo1.	Enhance T cell motility and improve tumor homing efficiency.
Effector phase	Reverse exhaustion	Inhibition/Degradation	1. Pharmacological Blockade: Using small molecule inhibitors (e.g., GsMTx4, Dooku1). 2. Gene Silencing: Utilizing siRNA/shRNA to knockdown Piezo1. 3. Protein Degradation: Developing Piezo1-targeted PROTAC degraders.	Alleviate mechanical inhibition, enhance cellular traction force and cytotoxic function.

### 4.2 B cells

#### 4.2.1 Piezo1 as a key initiator for B cell responses to membrane-bound antigens

In the physiological environment, many antigens exist in membrane-bound forms. B cells respond more robustly to such antigens than to soluble antigens. Research has revealed that this disparity stems from the differential capacity of B cells to sense mechanical signals. Upon contact with antigen-presenting substrates, whether flexible planar lipid bilayers mimic cell membranes or high-modulus rigid glass surfaces, the plasma membrane tension of the B cells increases. However, only rigid glass surfaces induce a substantial elevation in tension, sufficient to activate Piezo1, thereby triggering Ca²^+^ influx and cell spreading. Functional assays further corroborated that Piezo1 is a molecular prerequisite for B cell responses to membrane-bound antigens, but not soluble antigens. Using specific Piezo1 inhibitors, such as GsMTx4 or OB-1, or knocking down their expression with siRNA, significantly inhibits Ca²^+^ signaling and cell spreading triggered in B cells by membrane antigens, without affecting their response to soluble antigens (33). This finding defines Piezo1 activation as a critical early event in B cell activation by membrane-bound antigens.

Building on these findings, the research team further discovered that the high efficacy of human papillomavirus virus-like particle (HPV VLP) vaccines partly stems from their ability to mimic the physical properties of membrane-bound antigens, enabling processing by B cells via a Piezo1-dependent pathway. The research team conducted comparative studies using HPV VLPs and soluble HPV pentamers, which share identical chemical structures composed of the L1 protein but exhibit distinct physical properties; the former are spherical particles characterized by high stiffness, whereas the latter are soft soluble proteins. Specifically, comparisons between HPV VLPs and soluble HPV pentamers revealed that although both can be endocytosed by HPV-specific B cells and induce Ca^2+^ signaling, only the Ca^2+^ influx triggered by VLPs can be blocked by Piezo1 inhibitors, such as GsMTx4. Experiments using siRNA knockdown of Piezo1 confirmed that Piezo1 is crucial for efficient transport of HPV VLPs to LAMP1-positive MHC class II antigen-processing compartments. However, the transport of soluble pentamers to these compartments is unaffected by Piezo1. This indicates that Piezo1 function does not affect endocytosis per se, but rather determines the subsequent intracellular trafficking fate of the antigen. Moreover, the colocalization of HPV VLPs with MHC class II molecules is more prolonged and stable over time than that of soluble pentamers, which influences the subsequent antigen presentation and strength of the immune response (78). These findings provide a theoretical basis for the design of highly effective vaccines that mimic the physical characteristics of the membrane antigens.

#### 4.2.2 Piezo1 specifically regulates antibody class switching in B cells

Beyond its role in initial activation, Piezo1 is also involved in regulating the differentiation fate of B cells, particularly in antibody class-switch recombination. A key study found that Piezo1 exhibited significant isotype specificity, selectively enhancing TGF-β1-induced IgA class switching. This study demonstrated that the Piezo1 agonist Yoda1 specifically upregulated GLTα expression, promoted surface IgA expression, and increased IgA antibody secretion, whereas inhibition of Piezo1 function or knockdown of Piezo1 in mouse B cells impaired IgA production. Mechanistically, Piezo1 exerts this effect by enhancing the phosphorylation of Smad3, a downstream signaling molecule of TGF-β1. This indicates that Piezo1-mediated mechanical signals can crosstalk with key cytokine signaling pathways, precisely guiding B-cell differentiation toward an IgA-producing fate (22). Given that IgA is the primary antibody in mucosal immunity, the function of Piezo1 suggests its potentially important role in the establishment and maintenance of mucosal defense mechanisms.

We noted that the downstream TGF-β1-Smad3 pathway of Piezo1 appears to exert diametrically opposed effects in DCs and B cells. Although seemingly contradictory, this reflects cell-type-specific signal decoding mechanisms. In DCs, Piezo1 primarily acts as a negative regulator that controls the cytokine pool of TGF-β1. Piezo1 deficiency or inhibition leads to excessive secretion of TGF-β1 protein by DCs themselves; these increased ligands subsequently act on receptors on T cells or DCs themselves, indirectly resulting in enhanced Smad3 phosphorylation. Thus, the effect on DCs is ligand concentration-dependent (30). Rab5c, a key regulator of early endosomal trafficking, has been implicated in enhancing TGF-β receptor-mediated signaling pathways (79). In contrast, in B cells, Piezo1 does not directly alter the total secretion of TGF-β1 but instead directly optimizes receptor signal transduction via a Rab5c-dependent endocytic pathway. Upon Piezo1 activation, Rab5c is upregulated, promoting internalization and early endosomal trafficking of TGF-β receptors, thereby enhancing the efficiency of Smad3 phosphorylation at the receptor level. Consequently, the effect on B cells is dependent on receptor sensitivity (22).

Furthermore, the biological endpoints of the two regulatory modes are different. Dysregulation of the Piezo1-Smad3 axis in DCs primarily affects T cell differentiation, whereas activation of this axis in B cells specifically drives IgA class switching. This cell context-dependent disparity suggests that Piezo1 can convert identical physical stimuli into distinct immunological outputs, according to the functional requirements of the host cell.

#### 4.2.3 Potential role of Piezo1 in immune tolerance and autoimmunity

The function of Piezo1 extends beyond promoting immune activation, encompassing its complex roles in maintaining autoimmune tolerance and participating in autoimmune pathologies. Previous studies suggest that during B cell development, the mechanism distinguishing “self” from “non-self” may not only rely on antigen signal strength but also involve the integration of mechanical signals. Piezo1, potentially by sensing the physical properties of the immunological synapse, may assist B cells in discriminating between self-antigens and foreign antigens, thereby contributing to the establishment of tolerance (80).

However, under pathological conditions, this mechanical sensing mechanism can be disrupted. Based on investigations into the regulatory mechanisms of substrate stiffness on B cell activation, increasing the Young’s modulus of the antigen-presenting matrix from a lower level (e.g., 2.6 kPa) to a higher level (e.g., 22.1 kPa) induces significant activation disparities in B cells, even when recognizing identical antigen densities. Specifically, a high-stiffness microenvironment promotes a marked increase in the number of B cell receptors (BCR) and phospho-spleen tyrosine kinase (pSyk) microclusters within the B cell immunological synapse. Concurrently, the fluorescence intensity and volume of these signaling molecules were notably augmented, and the colocalization between BCR and pSyk was significantly enhanced. Ultimately, this stiffness-mediated signal potentiation leads to a significant upregulation of the B cell activation marker CD69 (81).

Furthermore, alterations in the microenvironmental stiffness influence the efficiency of antigen extraction by B cells. On stiffer antigen-presenting cell surfaces, B cells must rely on greater mechanical forces to extract antigens. This mode permits only high-affinity B cells to acquire antigens successfully, thereby enforcing stringent affinity-based selection. Conversely, on softer antigen-presenting cell surfaces, antigens are more prone to passive shedding, allowing low-affinity B cells to acquire antigens, which significantly improves the extraction efficiency but lowers the threshold for selection (82). In the context of autoimmunity, this stiffness-determined antigen extraction mode may compromise central and peripheral tolerance mechanisms, enabling aberrant activation of low-affinity autoreactive B cells.

Although no studies have directly reported the role of Piezo1 in antigen extraction, based on existing evidence, we reasonably speculate that Piezo1, functioning as a mechanosensitive ion channel, plays a vital role in B cell recognition of membrane-bound antigens and the sensing of physical signal changes mediated by microenvironmental stiffness (33, 80–82). Piezo1 likely enhances BCR-pSyk microcluster formation and signal amplification following antigen extraction, allowing self-antigens that would otherwise be insufficient to trigger robust activation in low-stiffness environments to surpass the B cell activation threshold within pathological high-stiffness microenvironments. This process potentially promotes the survival and activation of autoreactive B cells, thereby driving the inflammatory cycle. Consequently, this is a critical direction for future investigation.

## 5 Conclusion

This review synthesizes current evidence identifying Piezo1 as a key mechanosensor that integrates mechanical cues with immune cell functionality across diverse pathophysiological contexts. Research has demonstrated that Piezo1 extensively and precisely regulates the fate and function of diverse immune cells by mediating Ca^2+^ influx, and its effects exhibit high pathological context-dependency. In atherosclerosis, Piezo1 activation may drive proinflammatory polarization of macrophages to exacerbate disease progression, whereas during tissue repair processes, its activation can enhance phagocytic function to promote regeneration. Similarly, in T cells, Piezo1 serves as an essential molecule for initial activation; however, under persistent stimulation within the TME, it may conversely suppress T cell cytotoxicity.

The observed complexity and functional duality of Piezo1 underscore its context-dependent role in shaping immune outcomes, highlighting the importance of mechanical signaling in immune regulation. These context-specific effects present both opportunities and challenges for therapeutic intervention. Preclinical evidence suggests that targeting Piezo1 may offer a strategy for modulating immune responses via biomechanical pathways. For example, enhancing vaccine efficacy through matrix stiffening has shown potential in animal models. However, the challenge lies in translating these findings, which necessitates precise interventions. The challenge lies in translating these findings into clinical practice, which requires precise intervention as therapeutic efficacy is highly dependent on specific pathological contexts, cell types, and disease stages.

Future breakthroughs in this field will rely on addressing the core questions through multidisciplinary integration. This includes in-depth elucidation of the specific signaling networks of Piezo1 across different immune contexts, development of regulatory tools and targeted delivery strategies with cellular and spatiotemporal selectivity, and the establishment of reliable biomarker systems to guide clinical translation. In summary, advancing our understanding of Piezo1 provides critical insights into the mechanisms of mechanoimmunology, and identifies actionable targets for interventions in cancer, autoimmunity, fibrosis, and other pathologies.
